# Evaluating preservation methods for identifying *Anopheles gambiae s.s.* and *Anopheles arabiensis* complex mosquitoes species using near infra-red spectroscopy

**DOI:** 10.1186/s13071-015-0661-4

**Published:** 2015-01-27

**Authors:** Valeriana Simon Mayagaya, Alex John Ntamatungiro, Sarah Jane Moore, Robert Andrew Wirtz, Floyd Ercell Dowell, Marta Ferreira Maia

**Affiliations:** Ifakara Health Institute, P.O. Box 53, Ifakara, Tanzania; London School of Hygiene & Tropical Medicine, Keppel Street, WC1E 7HT London, UK; Swiss Tropical & Public Health Institute, Soccinstraße 57, 4002 Basel, Switzerland; University of Basel, Petersplatz 1, 4003 Basel, Switzerland; Centers for Disease Control and Prevention, Atlanta, GA 30329 USA; Engineering and Wind Erosion Research Unit, USDA ARS Centre for Grain and Animal Health Research, Manhattan, KS USA

**Keywords:** Near-infrared spectroscopy, *Anopheles gambiae s.s*, *Anopheles arabiensis*, Species identification, Preservation methods, Malaria

## Abstract

**Background:**

Near-infrared spectroscopy (NIRS) has been successfully used on fresh and RNA*later*®-preserved members of the *Anopheles gambiae* complex to identify sibling species and age. No preservation methods other than using RNA*later*® have been tested to preserve mosquitoes for species identification using NIRS. However, RNA*later*® is not the most practical preservative for field settings because it is expensive, requires basic laboratory conditions for storage and is not widely available in sub-Saharan Africa. The aim of this study was to test several cheaper and more field-friendly preservation methods for identifying sibling species of the *An. gambiae* complex using NIRS.

**Methods:**

In this study we describe the use of NIRS to identify sibling species of preserved *An. gambiae s. s.* and *An. arabiensis*. Mosquitoes of each species were placed in sample tubes and preserved using one of the following preservation methods: (i) refrigeration at 4°C, (ii) freezing at −20°C, (iii) drying over a silica-gel desiccant, (iv) submersion in RNA*later*® at room temperature, (v) submersion in RNA*later*® at 4°C, and (vi) submersion in RNA*later*® at −20°C. Mosquitoes were preserved for 1, 4, 10, 32 or 50 weeks before they were scanned.

**Results:**

Storage at 4°C was the only preservation method that, up to 32 weeks, did not result in significantly lower predicted values than those obtained from fresh insects. After 50 weeks, however, refrigerated samples did not give meaningful results. When storing for 50 weeks, desiccating samples over silica gel was the best preservation method, with a partial least squares regression cross-validation of >80%. Predictive data values were analyzed using a generalized linear model.

**Conclusion:**

NIRS can be used to identify species of desiccated *Anopheles gambiae s.s.* and *Anopheles arabiensis* for up to 50 weeks of storage with more than 80% accuracy.

## Background

In sub-Saharan Africa, mosquitoes of the *Anopheles gambiae* complex are the main vectors of malaria. Of the seven morphologically indistinguishable siblings of this complex, *An. gambiae* sensu stricto and *An. arabiensis* are the most effective malaria vectors and are widely spread throughout the continent, co-existing in many regions [1]. These two sibling species differ in their ecological and biological behaviors [2,3], and therefore their response to control measures is also different [4-7]. For this reason, species identification of this complex is critical for characterizing malaria transmission and for developing control strategies suitable to each ecological and social setting.

Near infrared spectroscopy (NIRS) is a tool that has the ability to differentiate siblings of the *An. gambiae* complex based on the composition of their biochemical components such as water, proteins, lipids and carbohydrates. NIRS is faster and cheaper than using polymerase chain reaction (PCR), the current gold standard [8], and it has been successfully used to identify the age and species of fresh and RNA*later*®-preserved *An. gambiae s.s.* and *An. arabiensis* [9-11]. Given the relative expense of RNA*later*® and that the use of NIRS on fresh mosquitoes is limiting in large-scale studies, other preservatives such as ethanol, desiccants (drierite and silica gel), refrigeration and Carnoy have been studied for preservation of mosquitoes for the purpose of age grading. Among these, desiccants (drierite and silica gel) and refrigeration appeared to be very promising in terms of accuracy and price [12]. Other than RNA*later*®, preservation methods have not been evaluated for identifying mosquito species using NIRS. It is important to identify a more affordable preservation method that allows for species differentiation from preserved specimens. This is critical for NIRS to become a feasible, low-cost option for researchers working on large-scale projects. The present study aims to test cheaper, more field-friendly preservation methods that can be used in conjunction with NIRS to identify the sibling species of *An. gambiae* complex, specifically *An. gambiae s.s.* and *An. arabiensis*.

## Methods

### Mosquitoes

*An. gambiae s.s.* and *An. arabiensis* were obtained from the Ifakara Health Institute (IHI) insectaries. *An. gambiae s.s.* were reared at 27°C and 80% relative humidity. *An. arabiensis* were reared inside a non-acclimatized insectary that was situated inside a semi-field system (screen house) and therefore subject to natural weather conditions [13]. Temperature and relative humidity were not monitored in the *An. arabiensis* colony. Larvae of both species were fed Tetramin fish food, and adults were provided with fresh 10% glucose solution daily. Mosquitoes were 4 days old and had not been previously blood fed.

### Preservation methods

*An. gambiae s.s.* and *An. arabiensis* were collected from the IHI insectaries. Approximately 45 mosquitoes were stored in 1.5 ml tubes for preservation by refrigeration at 4°C, refrigeration at −20°C and inclusion in RNA*later*®. To ensure better moisture absorption during silica gel desiccation, only approximately 20 – 25 mosquitoes were put in each 1.5 ml tube. A thin cotton layer separated the silica gel and the samples. The number of samples scanned for each species and preservation method are illustrated in Table 1.

**Table 1:** **The number of mosquitoes scanned for each species per preservation method**

**Preservation method**	***An. arabiensis***	***An. gambiae s.s.***
Fresh	134	134
Refrigeration at 4°C	533	543
Refrigeration at −20°C	666	673
Dried with silica gel and stored at room temperature	686	688
Stored in RNA*later*® at room temperature	672	673
Stored in RNA*later*® at 4°C	625	620
Stored in RNA*later*® at −20°C	668	663

accuracies across each storage duration and preservation method (Figure 1).

For each preservation method, mosquitoes were stored for 1, 4, 10, 32 or 50 weeks. For each storage time period and preservation method, three replicates were stored, with approximately 45 mosquitoes per replicate. For example, preservation by refrigeration for 1 week had 3 replicates, each containing approximately 45 mosquitoes.

### Mosquito scanning

Prior to scanning, fresh mosquitoes were anesthetized using chloroform vapors. Samples stored at 4°C and −20°C were allowed to reach room temperature before scanning. Those stored in RNA*later*® were first put on a paper towel to remove excess fluid. Using a LabSpec 5000 (ASD Inc., Boulder, CO) spectrometer as detailed previously [9], approximately 20 mosquitoes were placed on a 9 cm spectralon disc and immediately scanned, capturing only head-thorax. All spectra were collected using ASD RS^3^ software.

### Data analysis

ASD ViewSpecPro was used to convert spectra from ASD to GRAMS format (Thermo Galactic, Salem, NH). The GRAMS software PLSPlus/IQ was used to perform partial least squares (PLS) regressions and cross-validations on the spectra. For species identification, a cross-validation was calculated for a given preservation method using all three replicates for each storage-period. For analysis, *An. arabiensis* was assigned a value of 1, and *An. gambiae s.s.* was assigned a value of 2. All samples with a predicted value of less than 1.5 were classified as *An. arabiensis*, and above 1.5 as *An. gambiae s.s*. Six to 10 factors were used in cross-validation analysis. To avoid over-fitting the data, the number of factors used in regression coefficient plots were added up to the point where the plots of the regression coefficients were still interpretable (i.e., the positive and negative peaks are well identified along the plot). Data analysis was conducted as described previously [9]. To analyze the relationship between the prediction values for each preservation method versus fresh insects, a generalized linear model was fitted to a normal distribution, with preservation as the fixed effect and indicator variable. The dependent variable was the prediction value. A separate analysis was conducted for each storage-period of a particular preservation method compared to mosquitoes that had been scanned fresh and thus not subjected to a preservation method.

## Results

PLS cross-validation prediction accuracies (%) of the mosquito species *An. gambiae s.s.* and *An. arabiensis* for both fresh and preserved samples are reported in Table 2. Fresh samples had an average PLS cross-validation of 91.4% prediction accuracy (Table 2). In general, preservation by refrigeration at 4°C gave the best results for up to 32 weeks of storage (Figure 1). Desiccation over silica gel gave very good results for up to 50 weeks duration of storage, with a PLS cross-validation of consistently > 80% prediction accuracy. These predictions were significantly different from the predictions for fresh mosquitoes but still delivered over 80% accuracy. Preservation in RNA*later*® gave good results at 1 and 4 weeks of storage with a PLS cross-validation prediction accuracy of > 82%. However, results for samples preserved in RNA*later*® for 10, 32 and 50 weeks were poor when compared to the fresh predictions. Both *An. gambiae s.s.* and *An. arabiensis* showed similar prediction.

**Table 2:** **PLS cross-validation, % accuracy of species predictions using NIRS on preserved and fresh** ***An. gambiae s.s.*** **and** ***An. Arabiensis*** **samples**

**Storage duration (weeks)**	**Fresh**	**4°C**	**−20°C**	**Desiccated in silica gel**	**RNA** ***later*** **® room temp.**	**RNA** ***later*** **® at 4°C**	**RNA** ***later*** **® at −20°C**
*An. arabiensis*							
0	91.8	.	.	.	.	.	.
1		96.3	94.8	94.8	82.8	87.4	87.8
4		83.3	91.1	88.9	78.5	85.1	83.1
10		90.8	69.6	80.7	68.7	70.6	72.9
32		83.7	83.7	80.7	72.2	80.0	71.1
50		**	92.6	90.4	84.4	88.9	93.3
*An. gambiae*							
0	91.0	.	.	.	.	.	.
1		97.3	98.5	85.2	90.2	94.0	94.6
4		91.9	91.8	88.4	78.8	83.7	82.6
10		94.1	74.2	81.5	69.6	69.1	69.5
32		87.8	79.3	89.7	80.0	75.9	70.4
50		**	92.6	89.6	63.7	75.6	77.0
Average	91.4	90.7	86.8	87.0	76.9	81.0	80.2

Caption: **Samples refrigerated at 4°C for 50 weeks were not suitable for scanning.

**Figure 1:**
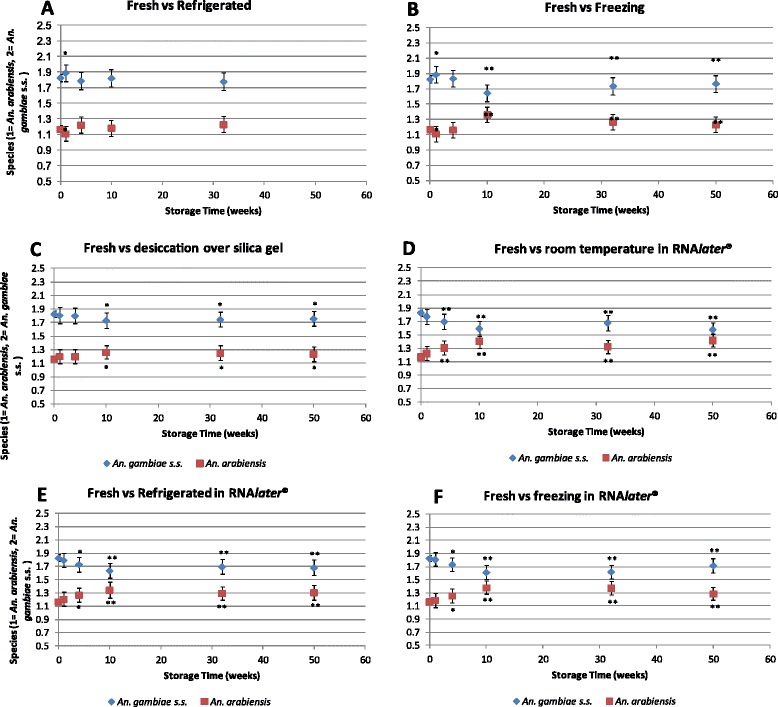
**This model estimated the means and standard errors obtained from a generalized linear model analyzing species prediction values obtained by near-infrared spectroscopy of preserved** ***Anopheles arabiensis*** **mosquitoes and** ***Anopheles gambiae s.s.*** Species are predicted to be *Anopheles arabiensis* if the prediction value is lower than 1.5 and predicted as *Anopheles gambiae s.s.* if the prediction value is higher than 1.5. Legend: * p-value <0.05; ** p-value < 0.001.

## Discussion

This study demonstrated that with varying degrees of accuracy depending on the preservation condition, NIRS can successfully be used to distinguish between laboratory-reared *An. gambiae s.s.* and *An. Arabiensis* that are preserved for up to 50 weeks. NIRS was capable of differentiating between laboratory-reared *An. gambiae s.s.* and *An. arabiensis* that had been preserved by refrigeration; dried over silica gel; and stored in RNA*later*®. Although the prediction values for mosquitoes dried over silica gel desiccant were significantly different from the prediction values for fresh samples, their PLS cross-validation predictions were always > 80% accurate up to 50 weeks storage duration. Thus, drying with silica gel desiccant was the best preservation method for long-term storage. The ability to distinguish between malaria vectors with an accuracy of 80% is ecologically relevant because it can be used to effectively plan and monitor vector control programs and to estimate the transmission risk that the known vectors can generate or sustain in the human population [14]. The results of refrigeration and desiccation over silica gel are in accordance with the findings obtained in a previous study where preserved *An. arabiensis* were successfully scanned for age prediction [12]. In that study, desiccation over silica gel (84.1% accurate) and refrigeration (81.3% accurate) gave the best results after 2 months of storage. This is an advantage because both species and age data can be obtained from the same mosquitoes preserved in silica gel desiccant or at 4°C.

In a study conducted by Dowell et al., refrigeration and desiccation over silica gel were among the cheapest preservation methods, where the cost of preserving samples in a 1.5 ml tube was $0.01 as compared to $1.00 for RNAlater® [12]. These methods allow for the preservation of many samples at a reasonably low cost relative to the cost of frequently transporting fresh samples from field sites to the location where the machine is stationed, or moving the machine to each study site. Although refrigeration and desiccation over silica gel are both comparably low in cost, the latter is more field-friendly. This is because silica gel desiccant is easy to handle and use in the field, and the only major requirement is to keep containers tightly closed to avoid moisture. Therefore, the preservation of mosquitoes with silica gel desiccant addresses the challenges posed by changes in tropical weather conditions and the instability of electricity, a common situation in much of sub-Saharan Africa.

The benefits of NIRS as a rapid [15] and non-destructive [9] method of identification of mosquito species is further cemented by the advantages of using mosquitoes preserved over silica gel desiccant (long lasting, cheap and field friendly), making the technique ideal for large-scale and long-term field studies. This method makes it possible to rapidly process many samples for both species identification and age grading at a low cost. Any method that allows for the collection, preservation and processing of more samples in a shorter time period than the conventional techniques of species identification and age grading, will allow for a larger sample size, which is essential for attaining more representative and meaningful study outputs.

Sample storage in RNA*later*® at 4°C and −20°C gave good PLS cross-validation results (>82%) for up to 4 weeks of storage, which confirms the results from previous NIRS studies on preserved mosquitoes [10,12]. The inconsistent RNA*later*® PLS cross-validation results at greater than 4 weeks and for all room temperature samples after 1 week and the sizable deviation from the prediction values for fresh samples, could mean that this preservation method is only suitable for species delineation for up to 4 weeks of storage when the samples are refrigerated or frozen. Additionally, RNA*later*® seems to be more appropriate for age grading studies than for species identification. Manufacturer specifications recommend that samples in RNA*later*® be stored for no longer than 1 week at 25°C, 1 month at 4°C, or indefinitely at −20°C [16]. Thus our RNA*later*® room temperature and 4°C results agree with the manufacturer recommendations. However, it is not clear why the RNA*later*® -20°C results beyond 4 weeks of storage were poor. Sometimes during storage mosquitoes do not stay immersed in RNA*later*® or become trapped in air bubbles. This could result in poor spectra and poor prediction results.

The findings of this study confirm the conclusions of the two previous studies [10,12] on the potential of using NIRS for species identification and age grading of preserved *An. gambiae s.s.* and *An. arabiensis*. With the ultimate goal of eliminating malaria, NIRS is a potential tool both for monitoring the disease and for evaluating interventions that involve age grading and species identification of *An. gambiae s.s* and *An. arabiensis*.

## Conclusions

Desiccation over silica gel appeared to be the most consistent and long-lasting preservation method. Previous studies have indicated that this method is cheap, long-lasting and field-friendly; therefore, we recommend using NIRS, a rapid and non-destructive technique, as the best method for species identification and age prediction. More focus should be put on this method for future field studies. Now that we know it is possible to use NIRS on preserved laboratory-reared mosquito samples, there is a need to validate NIRS to identify the age of field-collected mosquitoes. However, if NIRS is proven to accurately predict the age and species of wild *Anopheles gambiae* s.l., there will be a need to produce standard guidelines and operational procedures so this technology can be made widely accessible to scientists working in the field. Additionally, there is still a gap in knowledge regarding the individual characteristics of the mosquitoes that influence the NIRS absorption spectra and allow us to predict species and age. Pinpointing these particular characteristics could lead to the development of new methods, guaranteeing even higher prediction accuracies.
